# Exploring the experiences of operating room health care professionals' from the challenges of the COVID-19 pandemic

**DOI:** 10.1186/s12893-021-01437-3

**Published:** 2021-12-25

**Authors:** Fateme Mohammadi, Banafsheh Tehranineshat, Mostafa Bijani, Khodayar Oshvandi, Zohreh Badiyepeymaiejahromi

**Affiliations:** 1 Chronic Diseases (Home Care) Research Center and Autism Spectrum Disorders Research Center, Department of Nursing, Hamadan University of Medical Sciences, Hamadan, Iran; 2 2Research Center, Department of Nursing, School of Nursing and Midwifery, Shiraz University of Medical Sciences, Shiraz, Iran; 3 Department of Medical Surgical Nursing, Fasa University of Medical Sciences, Fasa, Iran; 4 Mother and Child Care Research Center, Hamadan University of Medical Sciences, Hamadan, Iran; 5 Department of Medical Surgical Nursing, Jahrom University of Medical Sciences, Jahrom, Iran

**Keywords:** COVID-19, Challenge, Operating room, Qualitative research

## Abstract

**Background:**

The operating room nurses have encountered several challenges during the current COVID-19 Pandemic, which subsequently impact their clinical performance. The present study aimed to investigate the experiences of operating room health care professionals' regarding the COVID-19 pandemic challenges in southern Iran.

**Methods:**

This is a descriptive qualitative study. The required data were collected using in-depth and semi-structured interviews. Accordingly, 19 operating room health care professions were included in this study through purposive sampling. The data were collected from February 2020 to August 2021 and then analyzed using the Granheim and Lundman’s method.

**Results:**

Three main themes and 9 sub-themes were presented in this study. They included: 1- Challenges of applying protocols and standards of care in the COVID-19 pandemic in the operating room (lack of specific guidelines regarding care protocols and standards for crisis management of Covid-19 in the operating room and impossibility of keeping social distance in the operating room). 2- Professional challenges (tedious and hard work, professional burnout, lack of adequate support by the managers and lack of personal protective equipment). 3-Psychological crises (indescribable anxiety and worry, parenting stress, and weakened resilience).

**Conclusion:**

Based on the findings of the present study, it was indicated that lack of a specific guideline in the COVID-19 pandemic led to a sense of indecision and confusion among operating room staff. In this regard, performing tedious tasks, feelings of tiredness and exhaustion, lack of support by the authorities, and lack of access to adequate facilities and equipment were reported as the professional challenges of the healthcare staff in the current COVID-19 pandemic, which consequently affected the quality of care and patient’s safety. It seems that hospital officials can use the results reported in this study for efficient planning and effective management during the COVID-19 pandemic.

## Introduction

The coronavirus pandemic with a highly increasing mortality rate is one of the main threats for the health status of human beings, especially professional health care personnel in the current century [1–3]. The World Health Organization introduced the treatment team staff as one of the most vulnerable groups during this COVID-19 pandemic century [4], due to the high risk of developing COVID-19 in hospitals. Moreover, despite taking precautions and controlling the infection, a large number of health care providers have been infected with the COVID-19 virus so far [5]. Accordingly, the American Operating Room Association declared that operating room staff during this COVID-19 crisis mostly endure high pressure and stress levels and face many challenges in care, which overshadow their professional performance. Accordingly, this can be due to the complex and stressful atmosphere in the operating room [6]. As the disease is progressing and the pandemic is developing, despite the current recommendations that surgery should be performed only in emergencies, the likelihood of positive COVID-19 emergencies for surgical intervention increases, which consequently leads to the high likelihood of contamination of the operating room environment [7]. In this context, Perrin (2020) states that in the operating room, where anesthesiologists, surgeons, and staff are accustomed to working in close contacts, keeping social distance would be challenging. Therefore, some factors such as non-observance of social distance between the staff and surgeons, lack of personal protective equipment, and emergency surgeries without corona diagnostic tests can create several challenges in the way of providing appropriate care. Intra-tracheal intubation, which can produce aerosols of airborne particles in the physically closed environment of the operating room, could also expose the staff and physicians to a high risk of coronavirus infection [8].

## Background in Iran

In Iran, the operating room staff includes surgeons, anesthesiologists, operating room nurses, and anesthesia nurses who are mostly experiencing lots of professional challenges and stresses in the current COVID-19 pandemic. Due to the fact that we are currently experiencing the fifth peak of the Corona pandemic in Iran, the health team is tolerating lots of professional and psychological pressures due to many challenges they are faced with. A number of operating room nurses and anesthesia nurses have been infected with Coronavirus and some have lost their lives. Awareness of the challenges and problems to which the operating room and anesthesia nurses are faced during COVID-19 crisis management is essential in designing management strategies to control the current infectious disease crisis. A previous review of the studies in this regard showed that few studies qualitatively explained the care challenges of operating room staff and physicians in the COVID-19 crisis. Therefore, to understand the concept and determine its dimensions deeply, performing a qualitative study is useful because such studies help to better explain the concept in the cultural context from the perspective of people who have a long-term involvement with that phenomenon [9]. In this design of research, the roles of individuals and their experiences are highly emphasized. Therefore, using a qualitative approach with a real understanding of the experiences can finally create a clear picture of these experiences [10].

In addition, providing quality care in the operating room is not possible unless by considering the rich experiences of the staff and physicians in the field of care challenges. Without awareness about the experience of operating room staff and physicians regarding emotions and difficulties in managing patients during crises caused by infectious and contagious diseases, it would not be possible to identify and provide appropriate solutions, thereby increasing the quality of care. Therefore, the present study aimed to investigate the experiences of operating room nurses regarding the COVID-19 pandemic challenges in southern Iran.

## Method

This is a qualitative research performed using a conventional content analysis approach. Conventional content analysis was also used when little information was available on the concept. Since the experiences of operating room staff and physicians regarding the challenges of COVID-19 crisis management in Iran are not fully understood yet, we made an attempt to use the mentioned approach. In reporting this qualitative study we have adhered to the COREQ (Consolidated criteria for reporting qualitative research) guidelines [11].

23 operating room health care professions were invited to participate in the study. However, 4 operating room health care professionals refused to participate due to their busy schedule and intensive shifts. Therefore, 19 participants (10 operating room nurses. 5 anesthesia nurses, 2 anesthesiologists, and 2 surgeons) from February 2020 and August 2021 were selected by purposive sampling. This is a technique widely used in qualitative research for the identification and selection of information-rich cases for the most effective use of limited resources. This involves identifying and selecting individuals or groups of individuals that are especially knowledgeable about or experienced in the phenomenon of interest. In addition to knowledge and experience, availability, willingness to participate, and the ability to communicate experiences are of importance.

The inclusion criteria were willingness to participate in the study and at least one year of work experience. Sixteen individuals were enrolled, and then semi-structured interviews were done to collect the data. Each interview lasted based on the participants’ willingness.

The participants were video called for interviews on WhatsApp at times which were convenient for them. The first author, who is an assistant professor of nursing and has carried out numerous qualitative studies performed and analyzed all the interviews. Interviews continued until data saturation. Saturation occurs when there is no new category emerging, and the categories are saturated based on their characteristics and dimensions [12].

The interviews started with general questions, including “Describe your first work experience with the COVID-19 disease crisis” and then continued by asking specific questions such as “Based on your experience, what challenges or job stresses are operating room staff faced with in the current COVID-19 pandemic? and “What solutions do you suggest for managing the COVID-19 crisis in the operating room Moreover, to increase the clarity of information, some probing questions such as “Can you explain more?", "What do you mean?", and “Can you give an example?" were asked. The interview process was followed considering the main purpose of this research. The interviews lasted for about 35 to 60 min. At the end of each interview, the author firstly listened to the interviews several times and then transcribed them on paper. Afterward, data analysis was performed simultaneously with data collection, immediately after each interview, and then the next interview was planned based on the findings of the previously performed interviews.

### Data analysis

Immediately after the first interview and simultaneous data collection, the data were analyzed using Granheim and Lundman’s method in 5 steps. First, the recorded speeches were transcribed; then, by carefully studying the manuscripts, the researcher tried to find the external and internal elements of the speech. In other words, immersion in the data was done to gain the participants' understanding first. In the nest stage, by re-reading the manuscripts, the semantic units were identified as sentences or paragraphs from the statements and texts of the interview. Fourth, the initial codes or open codes were extracted from it. In open coding, the text of each interview was read several times and the main sentences were extracted and recorded as codes. The related codes extracted based on similarity were then placed in subcategories. Finally, subcategories were merged to create the final themes [13]. For this purpose, the key points of the text were extracted as open codes by considering the overt and covert contents of the semantic units. Thereafter, these codes were classified based on their differences and similarities and the process of abstraction continued until the theme extraction themes [13]. The collected data were finally analyzed in MAXQDA.

### Trustworthiness

The accuracy and trustworthiness of the data obtained were tested using the criteria suggested by Guba and Lincoln [14]. In order to check the compliance of the findings with the experiences of the included participants, the extracted codes and classes were presented to three participants and their opinions were sought. Besides the participants of this study, the opinions of three individuals outside the study on the findings of the study were received as well in order to review the data analysis process and comment on its accuracy. To ensure transferability, the researcher tried to document all stages of the research, including data collection and analysis, coding and classification of codes, and the formation of the classes, so that it could be studied by other researchers in the future.

### Ethical considerations

The present study was conducted in terms of the principles of the revised Declaration of Helsinki, a statement of ethical principles that directs physicians and other participants in medical research involving human subjects. Prior to performing the interviews, all the subjects were informed about the objectives of the study, voluntary nature of their participation, data collection methods, reason for recording the interviews, role of the interviewer and the participants, and confidentiality and anonymity of the information. Subsequently, they were asked to sign the informed consent form if they were willing to participate. The participants were notified that they would be free to withdraw from the study at any time.

## Results

The means of the participants’ age and length of work experience were 35. 73 ± 5.42 years old and 9.52 ± 3.12 years, respectively. Other demographic characteristics of the participants are shown in Table 1.

**Table 1:** Descriptive characteristics of the participants

Participant	Age (years)	Gender	Education level	Specialty	Working experience (year)
P1	33	Female	Bachelor of Science	Operating room Nurse	10
P2	29	Female	Bachelor of Science	Operating room Nurse	4
P3	41	Female	Bachelor of Science	Anesthesia Nurse	17
P4	28	Male	Bachelor of Science	Operating room Nurse	3
P5	33	Female	Bachelor of Science	Operating room Nurse	9
P6	29	Female	Bachelor of Science	Anesthesia Nurse	3
P7	35	Male	Master of Science	Anesthesia Nurse	11
P8	31	Male	Bachelor of Science	Operating room Nurse	9
P9	40	Male	Master of Science	Operating room Nurse	16
P10	37	Female	Specialist	Anesthesiologist	4
P11	31	Male	Bachelor of Science	Operating room Nurse	8
P12	27	Male	Bachelor of Science	Anesthesia Nurse	3
P13	51	Female	Specialist	Surgeon	17
P14	26	Female	Bachelor of Science	Operating room Nurse	2
P15	39	Male	Specialist	Anesthesiologist	6
P16	33	Male	Bachelor of Science	Operating room Nurse	11
P17	45	Female	Specialist	Surgeon	12
P18	47	Male	Bachelor of Science	Anesthesia Nurse	17
P19	44	Female	Bachelor of Science	Operating room Nurse	19

COVID-19 crisis management challenges in the operating room led to the extraction of three themes and nine subthemes. Correspondingly, these three main themes included the challenges of applying the protocols and standards of care in the operating room, professional challenges, and psychological crises. Table 2 shows these themes and sub-themes.

**Table 2:** Themes and subthemes extracted from content analysis

Themes	Subthemes
Challenges of applying protocols and standards of care in the COVID-19 pandemic in the operating room	•Lack of specific guidelines regarding care protocols and standards for crisis management of Covid-19 in the operating room •Impossibility of keeping social distance in the operating room
Professional challenges	•Tedious and hard work •Professional burnout •Lack of adequate support from the managers •Lack of personal protective equipment
Psychological crises	•Indescribable anxiety and worry •Parenting stress •Weakened resilience

### Theme 1: challenges of applying protocols and standards of care in the COVID-19 pandemic in the operating room

The first theme was the challenges of applying protocols and standards of care in the Covid-19 pandemic in the operating room, which included lack of specific guidelines regarding the protocols and care standards in crisis management of COVID-19 in the operating room as well as the impossibility of social distance in the operating room.

#### Lack of specific guidelines regarding care protocols and standards for crisis management of Covid-19 in the operating room

One of the challenges of the operating room staff in managing COVID-19 in the operating room was found to be the lack of specific protocols and standards in the COVID-19 pandemic from the participants' point of view. Accordingly, from their perspectives, the operating room personnel are faced with a new care instruction every day, which leads to both uncertainty and confusion among the staff. In this regard, they do not really know which instructions to follow.

"There is no single, comprehensive guideline for operating room staff care standards regarding COVID-19. They want to apply the same instructions that have been developed for the staff of other wards of the hospital for the operating room staff, but the environment and working conditions in the operating room are very different from those of other wards. We come across a large number of instructions every day that have no efficiency. It is better to develop a single and specific instruction to be used" (Participant 8).

#### Impossibility of keeping social distance in the operating room

The second issue in the field of challenges of applying protocols and standards of care in the current COVID-19 pandemic in the operating room from the participants' point of view was found to be the impossibility of keeping social distance in the operating room.

"Operating room supervisors and clinical supervisors emphasize that you must keep your social distance, but they offer no solution. Is it possible for a team of staff and surgeons to work closely with the patient? I say stay away from each other and get away from the patient's bed. A moment of negligence and being away from the patient during surgery may endanger the patient's life." (Participant 17)

### Theme 2: professional challenges

The second major theme in this study was professional challenges, which includes tedious work, professional burnout, lack of adequate support, and lack of sufficient equipment.

#### Tedious and hard work

Heavy and tedious working in the operating room during the COVID-19 pandemic is one of the most important professional challenges for the operating room staff from the perspective of the participants, which was indicated to have negative impacts on the quality of care provided and patient’s safety.

"We really don't have energy left for us anymore. Working in the operating room is full of stress and in the COVID-19, This stress has increased a lot. In some situations, an operation lasts 8 to 10 hours, working in special clothes in COVID-19 condition in the operating room is very difficult and tedious, and the staff endures lots of stress and work pressures.” (Participant 7(

#### Professional burnout

From the participants' point of view, burnout is another professional challenge for operating room staff in the current COVID-19 pandemic*.*

“Unfortunately, in Iran, we have not managed the first wave yet, and we have immediately entered the second wave of the pandemic. Currently, we entered the fourth and fifth waves. All these hard works and stresses in this pandemic have led to the exhaustion of the staff and the treatment team, which consequently endanger their health status; a number of operating room staff lost their lives during the COVID-19 pandemic.” (Participant 12)

#### Lack of adequate support by the managers

One of the most important professional challenges in the COVID-19 pandemic from the perspective of operating room staff is the lack of adequate support by the managers. From these participants' point of view, during the period of the current COVID-19 pandemic, despite the fact that the staff are working hard while enduring a lot of stress, the managers do not provide enough support for them and even fail to provide their personal protective equipment.

“Staff in these difficult and complex situations do not receive support from the directors of the institution. Managers do not take any effective action to motivate and boost the staff’s morale. They even fail to pay their wages under this COVID-19 condition. They did not even give a letter of appreciation to the staff. They hold no meeting with the staff to listen to their problems in this difficult and exhausting situation.” (Participant 19)

#### Lack of personal protective equipment

Lack of personal protective equipment was another professional challenge for operating room staff during the COVID-19 pandemic.

"Operating room staff have mostly faced a shortage of personal protective equipment. Moreover, in some situations, a simple mask is not found in the ward. Face shields and special clothing are also scarce. Besides, the ventilation system in the operating room does not work well, so equipment providing better ventilation should be used. Many patients do not take the COVID test at all, but they are sent to the operating room. After the operation, we will find out that the patient's COVID test was positive. There must be facilities to perform a rapid COVID test, which is not available in our country unfortunately.” (Participant 10)

### Theme 3: psychological crises

The third major theme was psychological crisis, including indescribable anxiety and worry, parenting stress, and impaired resilience. From the perspective of the included participants, the operating room staff experiences various psychological crises during the current COVID-19 pandemic.

#### Indescribable anxiety and worry

Indescribable anxiety and worriedness were one of the psychological crises from the participants' points of view. Psychological stress due to having a direct contact with the infected patients and fear and anxiety about the high probability of this disease led to indescribable anxiety and worry among the staff.

“Operating room staff are experiencing numerous and severe psychological crises during the COVID-19 pandemic. Currently, in the fifth wave of the disease, these stresses and crises have doubled. We see our colleagues suffer from this disease, and in some cases our colleague has lost his/her life. These moments are very sad and have caused operation room staff to develop depression and anxiety disorders.” (Participant 18)

#### Parenting stress

Another psychological crisis from the perspectives of the participants was parenting stress.

“The COVID- 19 pandemic has had a profound effect on family functioning. We really do not know how to support and take care of our children in this COVID-19 pandemic, and we are all stressed and worried that we might pass this disease to our children. I have to take care of my child on my own. When I have to stay in the hospital for a few days due to the COVID-19 condition, I am always worried about my child who is at home.” (Participant 2)

#### Weakened resilience

Another psychological crisis described by the participants was the weakened resilience.

“We have no more patience and endurance; we have gone through five terrible and exhausting waves of the COVID-19 pandemic and endured lots of stress, so we are really tired and cannot continue tolerating this condition. All hospital managers tell us to be patient and tolerant; how can we tolerate this situation? How much really? How long?” (Participant 13)

## Discussion

This study aimed to explore the experiences of operating room staff regarding the challenges during the COVID-19 pandemic in southern Iran. Based on the obtained results, the challenges of operating room staff have emerged in the form of the following three main themes: challenges of using protocols and standards of care in the operating room, professional challenges, and job stress.

One of the main challenges for operating room staff was the application of the protocols and standards of care during the COVID-19 crisis. In this study, operating room staff reported that they experienced lack of specific guidelines on care protocols and standards for the management of COVID-19 crisis and the impossibility of keeping social distance in the operating room. However, in the study conducted by Nasiri (2020), the operating room staff kept their distance and had minimum contact with other staff and patients to maintain the safety of both staff and patients. Moreover, medical services were provided in the shortest time when the patient was in the operating room [15]. In the study carried out by Everson et al. (2021), operating room anesthesia nurses designed some protocols for caring the patients with COVID-19 and implemented them. As a result, training the operating room staff to use this protocol had reduced their confusion in dealing with patients infected with COVID-19 [16]. In India, the Minimum Manpower protocol was implemented in the operating room with minimal movement and mobility to prevent the transmission of contamination in the COVID-19 crisis [17]. Strict adherence to guidelines based on the evidence reported by the World Health Organization, Centers for Disease Control and Prevention, and personal and environmental hygiene was recommended to strengthen the infection control measures in the operating room [18]. In addition, to minimize the occupational risks, more innovations and innovative measures are needed to prepare the staff [19]. Furthermore, hospitals should develop their own internal protocols based on those of WTO and then hold adequate training programs for their staff involved in surgery and caring for the patients infected with Coronavirus to avoid any confusion and uncertainty among the operating room staff in the current COVID-19 crisis. Additionally, all the staff should perform their tasks according to the guidelines set for the ward.

The professional challenges experienced by operating room staff were among the other findings of the present study, which included tedious work, burnout, lack of adequate support, and lack of adequate equipment. In a study conducted in Iran, stressful work environment, doubts on the occurrence of unexpected conditions, lack of support received from officials, and lack of adequate equipment were among the challenges experienced by the nurses who provided healthcare to the patients infected with COVID-19. In addition, staff shortage and lack of equipment were indicated to affect the quality of nursing care [20].

The results of some other studies also showed that most of the problems experienced by nurses during caring for and dealing with COVID- 19 patients were as follows: staff shortage, fear of getting infection, and excessive fatigue due to long working hours without any proper nutrition [21, 22]. Lack of equipment and facilities, like PPE (Personal Protective Equipment), was also reported as one of the challenges raised by professional caregivers during caring a patient infected with COVID-19 [23, 24]. Bianco et al. (2020) also stated that protecting the operating room personnel was crucial in prevention of getting infected with COVID-19. In this regard, they are recommended that they should use personal protective equipment such as Aerosol Box [25].

Therefore, to improve the quality of nursing care and health services in the current COVID-19 pandemic, the hospital officials are recommended to pay more attention to the identification of the staff’s needs and providing adequate resources and facilities. In this regard, it is important to provide adequate manpower and support to officials through paying attention to the staff’s nutrition, organizing their working hours, providing adequate protective equipment, holding stress management training sessions, and providing psychological and medical support to them.

Another finding of the present study was the psychological crises of the operating room staff, which included their indescribable anxiety and worry, parenting stress, and impaired resilience in the COVID-19 crisis. In the carried out study by Nasiri (2020), the operating room staff reported that they experienced several mental conflicts during dealing with patients infected with COVID-19. All the participants expressed fear and concern about their infection and that of their family and relatives. Having small children and elderly parents with underlying diseases in the family added to their stress and anxiety. Among the main causes proposed for having the fear of COVID-19, unknown and complete lack of knowledge about coronavirus, rapid spread of lethality, and lack of vaccine and definitive treatment for this disease were noted as well [15].

The results of previous studies performed in Iran showed that nurses in COVID ward mostly experienced high anxiety. Based on the participants' experiences, Corona anxiety leads to fatigue, decreased concentration, sleep disturbance, decreased resilience, and burnout; also, it threatens the nurses' mental safety [17–20]. Also, Felice et al. (2020) in Italy stated that healthcare workers, especially women who work in high-risk wards, reported an increase in workload and the need for more psychological support during the COVID-19 pandemic [26].

According to the results of a study conducted during the COVID-19 pandemic, being a parent adds to the individuals’ stress. In this regard, workplace stress, fear of transmitting the infection to their children, necessity of staying in the hospital to stay away from their children, and inability to monitor the upbringing of children were the mentioned issues that have caused the nurses to experience excessive stress and consequently fatigue and burnout 7 [27].

In another study, nurses were overworked and worried that their children and family members would be infected with this disease. Therefore, besides feeling tired due to a great deal of work as well as fear and worry about endangering their health and families, they have suffered from high anxiety and stress levels [28]. Therefore, keeping nurses up-to-date by providing sufficient and comprehensive information on COVID-19 and ensuring that PPE is available may subsequently help us to reduce the staff’s fear and anxiety [29]. In brief, crisis management training for nurses, rooting out their stress and anxiety, assessing the health statuses of the staff, and reassuring them about the provision of essential first aids and psychological support needed help the m to prevent their psychological crises.

### The limitations of the study

One of the limitations of the present study was that the interviews were individually conducted only with operating room and anesthesia staff. Therefore, performing more focused interviews and also interviewing with other members of the operating room team, such as surgeons and anesthesiologists, can provide richer information in this regard.

### The strengths of the present study

To the best of our knowledge, this is the only study performed with a qualitative approach in Iran, aiming to explore the experiences of the operating room staff regarding the challenge of COVID-19 in the operating room, which is considered as a type of innovation. In addition, this study has not been done in other countries with a qualitative approach, or if done, it is very limited.

## Conclusion

Based on the findings of the present study, it was indicated that lack of a specific guideline in the COVID-19 pandemic led to a sense of indecision and confusion among operating room staff. In this regard, performing tedious tasks, feelings of tiredness and exhaustion, lack of support by the authorities, and lack of access to adequate facilities and equipment were reported as the professional challenges of the healthcare staff in the current COVID-19 pandemic, which consequently affected the quality of care and patient’s safety that have severely affected their professional performance and mental health. Therefore, health system managers and policy- makers can use the results of this study for efficient planning and effective management of operating room conditions during the COVID-19 pandemic and take key steps to reduce the challenges and needs of the staff in the COVID-19 crisis. Accordingly one of the priorities of the administrators of operating room centers under these conditions should be preserving the psychological health of the personnel who are dealing with COVID-19 patients. Development of evidence-based operating room care protocols and educational programs for raising the personnel’s awareness and facilitating their adaptation to the challenging conditions of the COVID-19 crisis will improve the quality of operating room care services.

## Data Availability

The datasets used and/or analysed during the current study are available from the corresponding author on reasonable request.

## Abbreviations

WHO: World Health Organization
COVID-19: Coronavirus disease 2019
EMS: Emergency medical services
PPE: Personal protective equipment
PTSD: Post-traumatic stress disorder
